# Microclimate monitoring of Ariadne’s house (Pompeii, Italy) for preventive conservation of fresco paintings

**DOI:** 10.1186/1752-153X-6-145

**Published:** 2012-11-28

**Authors:** Paloma Merello, Fernando-Juan García-Diego, Manuel Zarzo

**Affiliations:** 1 Department of Applied Physics (UD Agriculture Engineering), Universitat Politècnica de València, Camino de Vera s/n, 46022, Valencia, Spain; 2 Valencian Institute for Conservation and Restoration of Cultural Heritage (IVC+R), C/ Genaro Lahuerta 25-3º, 46010, Valencia, Spain; 3 Center of Physical Technologies, Associated Unit ICMM-CSIC/UPV, Universitat Politècnica de València, Av. de los Naranjos s/n, 46022, Valencia, Spain; 4 Department of Applied Statistics, Operations Research and Quality, Universitat Politècnica de València, Camino de Vera s/n, 46022, Valencia, Spain

**Keywords:** Multivariate monitoring, Temperature and relative humidity sensors, Cultural heritage, Preservation of open-air frescoes

## Abstract

**Background:**

Ariadne’s house, located at the city center of ancient Pompeii, is of great archaeological value due to the fresco paintings decorating several rooms. In order to assess the risks for long-term conservation affecting the valuable mural paintings, 26 temperature data-loggers and 26 relative humidity data-loggers were located in four rooms of the house for the monitoring of ambient conditions.

**Results:**

Data recorded during 372 days were analyzed by means of graphical descriptive methods and analysis of variance (ANOVA). Results revealed an effect of the roof type and number of walls of the room. Excessive temperatures were observed during the summer in rooms covered with transparent roofs, and corrective actions were taken. Moreover, higher humidity values were recorded by sensors on the floor level.

**Conclusions:**

The present work provides guidelines about the type, number, calibration and position of thermohygrometric sensors recommended for the microclimate monitoring of mural paintings in outdoor or semi-confined environments.

## Background

Pompeii was a village of ancient Rome located about 26 km southeast of the modern city of Naples (Italy). Around the year 62 AD, an earthquake severely damaged Pompeii and other nearby locations. Since then, the city was rebuilt until the eruption of Mount Vesuvius in 79 AD. Pompeii at that time had a population of about 15,000 inhabitants. The violent eruption of this volcano buried the city and preserved the ruins for centuries. Thick layers of ash covered Pompeii and Herculaneum, both towns placed at the base of Mount Vesuvius. Their names and locations were forgotten until the 18^th^ century when a new interest for antiquity led to excavations [1]. Herculaneum was rediscovered in 1738 and Pompeii in 1748. Since then, a campaign was launched to unearth both cities, revealing many intact buildings and valuable wall paintings.

Pompeii offers a picture of Roman life in the first century. The forum, baths, many houses, and some villas remained in a surprisingly good state of conservation. The site was declared World Heritage by UNESCO in 1997 and has become a popular tourist destination, with 2.5 million visitors in 2007. Pompeii is an open-air museum of 1,500 buildings comprising around 20,000 m^2^ of mural fresco paintings [2]. Pompeian interior frescoes have become a main source of knowledge about Roman painting up to 79 AD, not because the city was so important at that time but because its tragic end has preserved in good conditions all its wall frescoes for posterity to study [3].

An imposed moratorium stopped excavations in the site to focus the efforts in maintaining the unburied ruins and leaving the remaining excavations for future generations. At present, the access to the ruins is more restricted for tourists, and less than one third of the houses open in the 1960s are currently available for public visits. This is due to the endless maintenance works to prevent the deterioration of buildings already unearthed [4]. Apart from ruin fallings, at least 150 m^2^ of fresco paintings and plaster works are lost every year due to lack of maintenance.

### Description of Ariadne’s house

Ariadne’s house is a Pompeian *domus* (i.e., a single-family house owned by the upper classes) situated in a privileged location at the city center (Regio VII, *insula* 4). It is a *domus* of Hellenistic inspiration with an extension of 1,700 m^2^. The elevation above sea level of Pompeii is about 14 m. The prevailing wind directions are northeast (October to February) and south (March to September), with an average wind speed of approximately 2.57 m/s (http://es.windfinder.com). The house was built at the end of the second century BC and was heavily damaged during the earthquake of 62 AD, not having been finished its reconstruction on the fateful date of Mount Vesuvius eruption [1]. Most inner walls of the house were originally decorated with frescoes. The complete pictorial collection of Ariadne’s house has a remarkable quality and was probably created by the same artists’ team.

**Figure 1:**
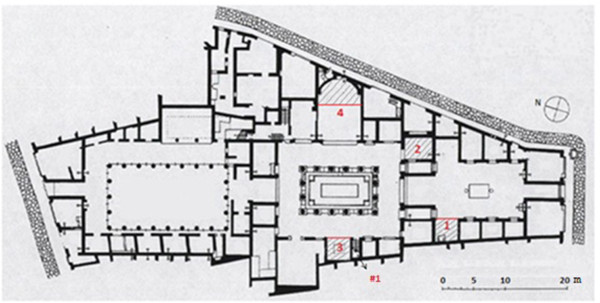
**Plan of Ariadne’s house.** Lodgings marked as 1 to 4 are the only roofed ones (parallel tilted lines delimit the covered area). Data-logger #1 was located on the top of an outside wall next to room 3 at 3 m from the ground level and it was covered with a ceramic tile.

Pompeii has been preserved in an enviable conservation state under the layers of ash, but most buildings suffered serious damages during the volcanic eruption and the majority of roofs came down. An aerial view (http://maps.google.es) of Ariadne’s house (40° 44' 57.77" N, 14° 29' 14.37" E) and nearby buildings reveals that they are well preserved but the roof is lacking in most of them. This is the case of Ariadne’s house, with only one room (marked as 4 in Figure 1) partly covered with a roof of ceramic tiles (14 m^2^) that was settled in the 1950s. After a preventive actuation in the 1970s, roofs made of transparent polycarbonate sheets supported by metallic structures were established in three rooms of the house (1, 2 and 3 in Figure 1) in order to protect the frescoes inside from rainwater. During the following decades, mural frescoes in all rooms that had been left uncovered were seriously damaged due to direct contact with rainwater and the unfavorable thermohygrometric conditions mainly during the summer [1].

Room 2 is comprised by four walls, the northwest one with a large window and a doorway in the SW wall. As a result, the indoor microclimate is more isolated from the outside environment compared with the other rooms under study that are delimited by three walls. Rooms 1 and 3 are open to the courtyard by their NE side. Room 4 is open to the atrium by its west side. It is the largest one and has only one wall decorated with frescoes that presents a semicircular shape (Figure 1).

An extensive scientific literature is available about the analysis of materials and characterization of mural paintings in Pompeii [5-8] and other Roman cities [9,10]. In the benchmark of a conservation project started in 2008, a series of works were carried out in Ariadne’s house: microclimate monitoring, electromagnetic radiation measurements, study of materials, and photographic report. The purpose was to assess the conservation state of frescoes and the most convenient future restoration works of Ariadne’s lodgings still containing mural paintings. These were all covered rooms (1 to 4 in Figure 1), whose roofs have preserved wall frescoes during the last decades.

The multidisciplinary team has studied the problems of salt efflorescence, atmospheric pollution and other pathologies associated with the materials in order to determine the causes of fresco degradation and propose conservation actions [11]. It was found that all pigments are from inorganic origin. The deterioration of frescoes due to inappropriate temperatures in the rooms assessed by thermography was also discussed. The multidisciplinary approach has been fundamental to provide the basic guidelines for future in-depth restoration works. Regarding the microclimate study, a set of 26 thermohygrometric probes comprised by temperature and relative humidity (RH) data-loggers were installed for monitoring ambient conditions inside the roofed rooms. The statistical analysis of data recorded by these sensors will be the subject of the present research.

Different reported studies have measured air temperature, RH and other parameters inside museums for the preventive conservation of their collections [12-15]. Similar works have monitored the indoor environment in churches, as they contain valuable artifacts [16-21]. However, very few studies have characterized ambient conditions of outdoor archaeological sites [22-24] or semi-confined environments [25]. The present work reports for the first time a microclimate research conducted in the ruins of Pompeii, which is of relevant interest because inappropriate conditions of temperature and RH are causing severe damages on the valuable fresco paintings. Moreover, results reported here will provide guidelines to establish thermohygrometric monitoring systems in similar open-air archaeological sites for preventive conservation.

### Experimental

#### Description of data-loggers

The use of autonomous devices was the best option in this case due to the lack of electric supply in Ariadne’s house. Each RH data-logger (Datalog Hygrochron DS1923) contains a humidity sensor with an accuracy of ± 5% [26]. Although this model can also record temperatures, it was decided to use independent devices (Datalog Thermochron DS1922L) for the temperature monitoring [27], which has the same accuracy (±0.5°C) as DS1923. The reason was to expand the data storage capacity of the monitoring system.

A set of 52 data-loggers, 26 of each model, were purchased directly from the manufacturer (Maxim Integrated Products, Inc., Sunnyvale, CA) and they were calibrated prior to their installation as described ahead. These devices resemble button-like batteries, with 17.4 mm of diameter and 5.9 mm of height. Given their small size, each pair of DS1923 and DS1922L data-loggers were assembled together by means of a metallic structure and will be referred to hereafter as thermohygrometric probe. This probe is isolated by an external PVC structure of dark-gray color, which allows a convenient fixing to the wall, and has a cylindrical shape with a diameter of 6 cm. It is filled with polyurethane foam to isolate the data-loggers from the PVC structure. The assembly allows both data-loggers to be directly in contact with the ambient air, and therefore it was assumed that the supporting structure had a negligible effect on the recorded data.

## Results and discussion

### Calibration of sensors

**Figure 2:**
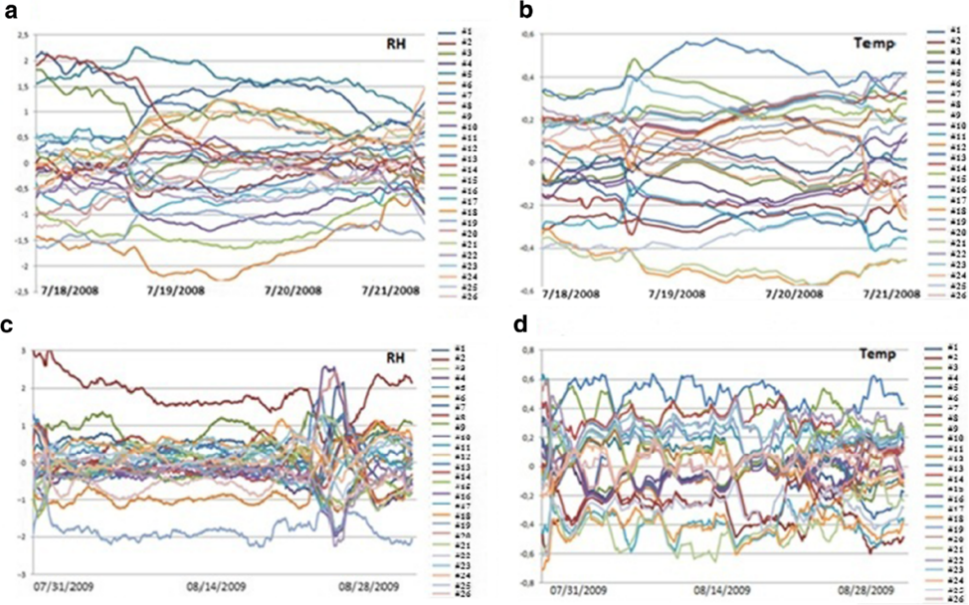
**Calibration of RH and temperature sensors.** Difference between RH and temperature recorded by each probe with respect to the average from all probes in two different calibration periods: first, from 18^th^ to 21^st^ July 2008 (**a:** RH; **b:** temperature) or second, from July 31^st^ to August 28^th^ 2009 (**c:** RH, **d:** temperature). Trajectories were smoothed using a moving average with a window size of one day.

The time series of temperature or RH recorded by one data-logger reflects the parameter evolution along the time, and it is often so-called as trajectory. Figure 2 shows that the average time series were slight in the calibration experiment. As all trajectories are quite parallel, the average registered during the calibration period by each data-logger is a representative value. If average temperatures obtained in the first period are compared with those from the second calibration stage, it turns out that the correlation is statistically significant (r = 0.934, p <0.0001). This result implies certain bias that should be corrected. For the first period, the bias was calculated as the mean temperature recorded by each data-logger minus the average from all of them, which was regarded as the exact value. The same procedure was applied for the second period. Next, the mean of both bias estimations was calculated (shown in Table 1) resulting a range from −0.44°C (#18) to +0.53°C (#13), which is consistent with the measurement error of ±0.5°C indicated by the manufacturer [27].

**Table 1:** **Height and bias**^**a**^**of temperature and RH data-loggers**

**Code**	**Height**^**b**^	**T**_**bias**_	**RH**_**bias**_	**Code**	**Height**^**b**^	**T**_**bias**_	**RH**_**bias**_
#1		−0.015	0.647	#14	0	0.405	−0.096
#2	185	−0.321	0.425	#15	30	0.175	−0.351
#3	290	−0.088	0.714	#16	0	0.027	−0.532
#4	153	−0.119	−0.365	#17	338	−0.298	−0.084
#5	0	0.018	0.760	#18	300	−0.438	0.495
#6	0	0.064	−0.208	#19	310	0.206	−1.506
#7	163	−0.168	0.372	#20	290	0.035	−0.198
#8	0	−0.204	0.342	#21	175	−0.424	0.464
#9	189	0.305	0.059	#22	240	0.291	−0.555
#10	240	−0.029	−0.550	#23	330	0.224	0.613
#11	0	0.199	0.204	#24	117	0.010	0.308
#12	210	−0.115	−1.112	#25	54	−0.304	0.233
#13	0	0.529	0.435	#26	15	0.036	−0.512

^a^Temperature bias (T_bias_ in °C) and relative humidity bias (RH_bias_ in %), averaged for both calibration periods (codes as in Figure 17).

^b^Distance to the floor level (cm).

Probe #2 was the one with highest RH values recorded in the second period (Figure 2c, bias = 1.9%), but it was the third one with lowest RH in the first stage (Figure 2a, bias = −1.1%). Thus, from the first to the second period, the shift is 1.1+1.9 = 3%. This error seems too high as the purpose of the present study is to discuss the slight differences recorded among sensors, and no bias correction was applied to #2. This abnormal performance was caused by an accidental water drop over this data-logger during its uninstallation. If RH data from #2 are disregarded for the calibration study, it turns out that the correlation between the average RH recorded by each data-logger in the first *vs.* the second calibration period is statistically significant (r = 0.701, p = 0.0001). Again, this result reveals certain bias, and the same procedure described for temperature was applied to estimate the errors. The resulting bias estimations range from −1.5% (#19) to +0.8% (#5). This range is much lower than the measurement error of ±5% indicated in the manufacturer data sheet [26]. It was decided to correct all temperature and RH values collected in Ariadne’s house according to the estimated biases (Table 1) in order to improve the reproducibility of the monitoring system.

In order to determine more accurately the real bias, a further experiment was conducted with aqueous solutions of two salts. Results confirmed that the bias was small and irrelevant for the purpose of the present study.

### Descriptive data analysis of mean trajectories

Knowledge about ideal or limit values of microclimate parameters for conservation of cultural heritage is still poor [28]. The Italian UNI 10829 [29] and DM 10/2001 [30] are currently the approved standards on this issue. They do not provide guidelines for the conservation of outdoor paintings, but nevertheless the admissible values of RH and temperature indicated by both standards can be taken as a reference for the conservation of Pompeian frescoes. According to [29], the recommended range of RH and temperature for mural paintings is 55 – 65% and 10 – 24°C, respectively. These ranges are narrower than the ones suggested by [30]: 45 – 60% and 6 – 25°C. Taking into account that both standards were developed for indoor conditions, we decided to consider here the intervals given by [30], which are wider and could be better extrapolated for outdoor conditions.

**Figure 3:**
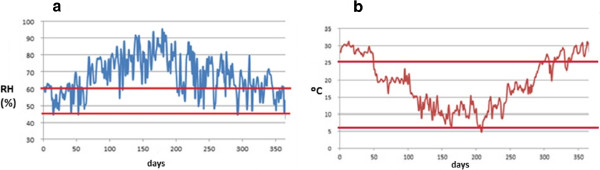
**Smoothed time series of RH and temperature.** Average RH (**a**) and temperature (**b**) recorded by all probes (except #1) during one year (day 0 corresponds to July 23^rd^ 2008). The time series was smoothed using a moving average with a window size of one day. Horizontal lines in red correspond to the ranges recommended by the standard DM 10/2001 for the conservation of mural paintings.

**Figure 4:**
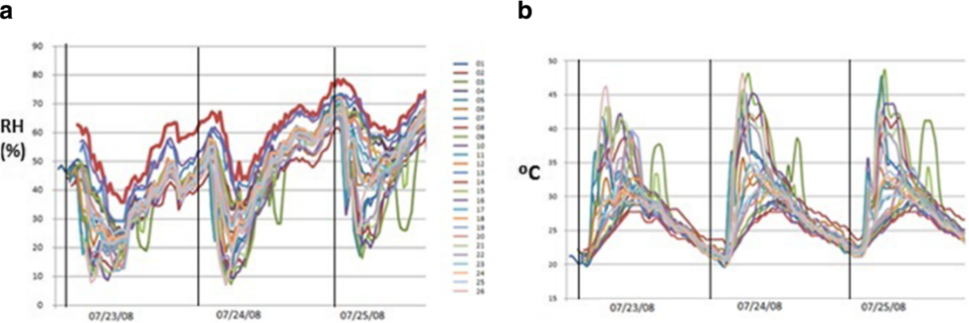
**Trajectories of RH (a) and temperature (b) recorded by all probes during three summer days (23**^**rd**^**to 25**^**th**^**July 2008).** Vertical lines correspond to midnight (0:00 hr).

Figure 3 shows the evolution of RH and temperature recorded by all probes in a period of one year. It turns out that the mean RH was out of the range recommended by the standard in 272 days during one year, while the mean temperature was higher than 25°C in 116 days. Thus, ambient conditions are too humid in winter and too hot in summer for the preservation of fresco paintings. The conservation risk is even more serious taking into account the marked daily variations of temperature and RH (Figure 4) and given that high peaks of temperature can be reached in summer. Actually, 31.2% of all recorded temperatures were above 25°C, and 73.1% of RH data were above 60%.

Daily oscillations of temperature and RH in summer can be up to 25°C and 40%, respectively (Figure 4). High variability is inappropriate for the conservation of fresco paintings, which encourages some kind of corrective action to reduce the temperature at least in summer [30]. A monitoring system will be necessary to study the effectiveness of any action of this kind, and to assess the risks for the long-term conservation of frescoes.

Figure 4a shows that all RH trajectories are quite parallel. Thus, the average RH of all values recorded by each sensor during the monitoring study characterizes the basic dissimilarities among RH time series. In the case of temperature, not all trajectories follow the same pattern. Consequently, the average value is not enough to describe conveniently the differences among temperature trajectories, and additional parameters were studied using bivariate plots and analysis of variance as described below.

### Detection of outliers

**Figure 5:**
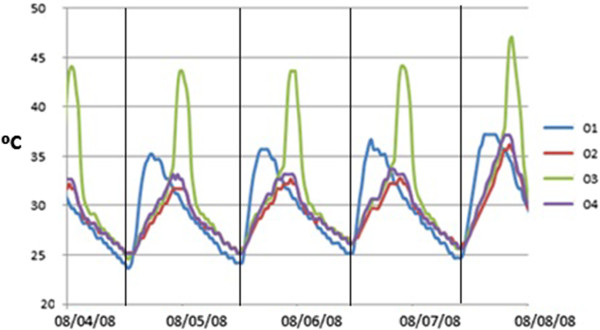
**Temperatures in room 4.** Temperatures recorded by data-loggers in room 4 (#2, #3 and #4) during four summer days (4^th^ to 8^th^ August 2008). Values of #1 are also shown for comparison.

**Figure 6:**
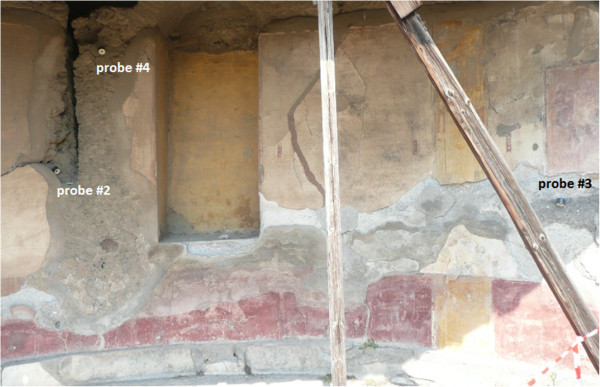
**Location of probes #2, #3 and #4 in room 4.** The picture was taken at 1:05 PM. The brighter zone at the lower right corner corresponds to sunlight incident on the frescoes that affects #3 from 2:00 PM to 8:00 PM.

Certain atypical shapes are observed in Figure 4b. Thus, prior to comparing average values, it is convenient to discuss those trajectories with abnormal deviations from the average temperature recorded by data-loggers in the same room. For this purpose, all trajectories were carefully inspected. Figure 5 shows that probe #3 recorded an abnormal increase of temperature at about 2:00 PM, and recovered the common pattern at 8:00 PM approximately. By contrast, in the same time frame, its RH time series underwent a sudden decrease (Figure 4a). The reason seems to be an effect of direct sunshine radiation that heats the data-logger during this time period as reflected by Figure 6.

In order to check if the atypical peaks of temperature recorded by #3 occurred all the year round, we calculated the differences between #3 and #4. The latter was taken as a reference data-logger in the same room with a normal performance. Next, the maximum value of this difference was calculated for the 48 time observations corresponding to each day: MAX $\left[\left(temp_{t_{1}}^{\text{\textbackslash{}\#3}}-temp_{t_{1}}^{\text{\textbackslash{}\#4}}\right)\ldots \left(temp_{t_{48}}^{\text{\textbackslash{}\#3}}-temp_{t_{48}}^{\text{\textbackslash{}\#4}}\right)\right]$.

**Figure 7:**
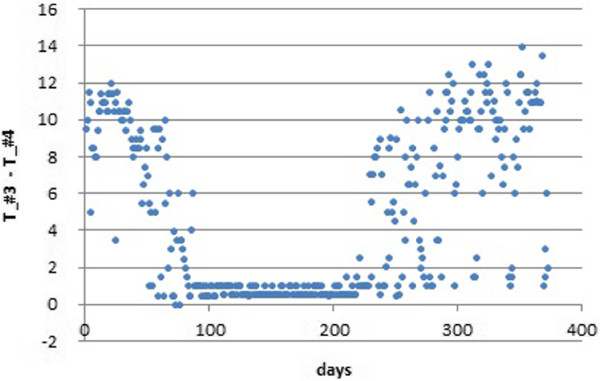
**Maximum daily differences of temperature between data-loggers #3 and #4.** Calculated for the 372 days of the period under study (day 0 corresponds to July 23^rd^ 2008).

If this daily difference is plotted for the period under study (Figure 7), it turns out that values are close to zero (i.e., both data-loggers presented very similar trajectories) from October 12^th^ 2008 to February 23^rd^ 2009. Out of this period, however, the differences are remarkable. The reason seems to be that no direct sunlight reached data-logger #3 during this particular period. The abnormal temperature increase of #3 did not occur in a few days of spring and summer probably due to the presence of clouds or rainy conditions that reduced sunshine.

**Figure 8:**
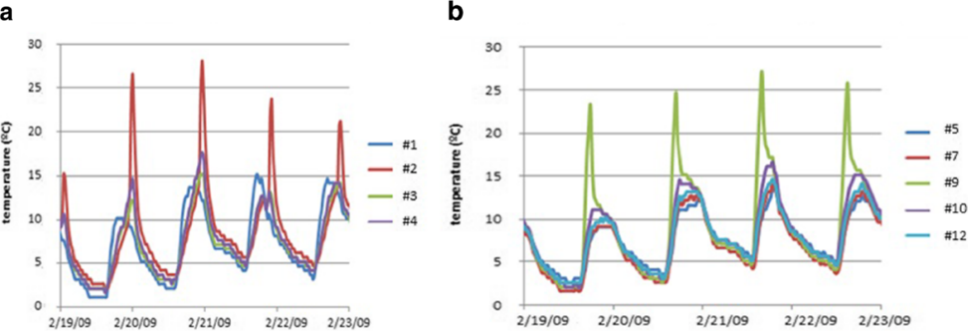
**Abnormal peaks of temperature recorded in room 4 and 3.** Temperatures recorded by probes in room 4 (**a**) and five probes in room 3 (**b**) during four winter days (19^th^ to 23^rd^ February 2009). Values from #1 are also included for comparison.

Figure 8 shows abnormal daily peaks of temperature recorded by #2 that occurred in winter from 2:30 PM to 6:30 PM approximately. Similarly, the temperature time series of #9 (room 3) also reflects sudden peaks (Figure 8b) in winter from about 8:30 AM to midday. In the same time frames, both data-loggers registered an atypical decrease of RH (Figures not shown). Again, sunlight incident on these particular sensors seems to be the reason. The time series of #1 did not deviate from the common pattern in summer (Figure 5) nor winter (Figure 8a) probably because this probe was covered with a tile and never received sunlight.

The sudden variations observed in certain trajectories at particular day frames can be seen as a mistake in the experimental setup. This is a problem for the statistical analysis, and the abnormal data of probes #2, #3 and #9 were discarded after carefully checking the values. Of course, direct sunshine on sensors produces abnormal temperature variations that should be avoided, but in long-term monitoring it is not evident how to determine the different trajectories of solar radiation incident on all walls during the year. This is an important recommendation for future studies of outdoor ambient monitoring.

### Daily mean trajectories

**Figure 9:**
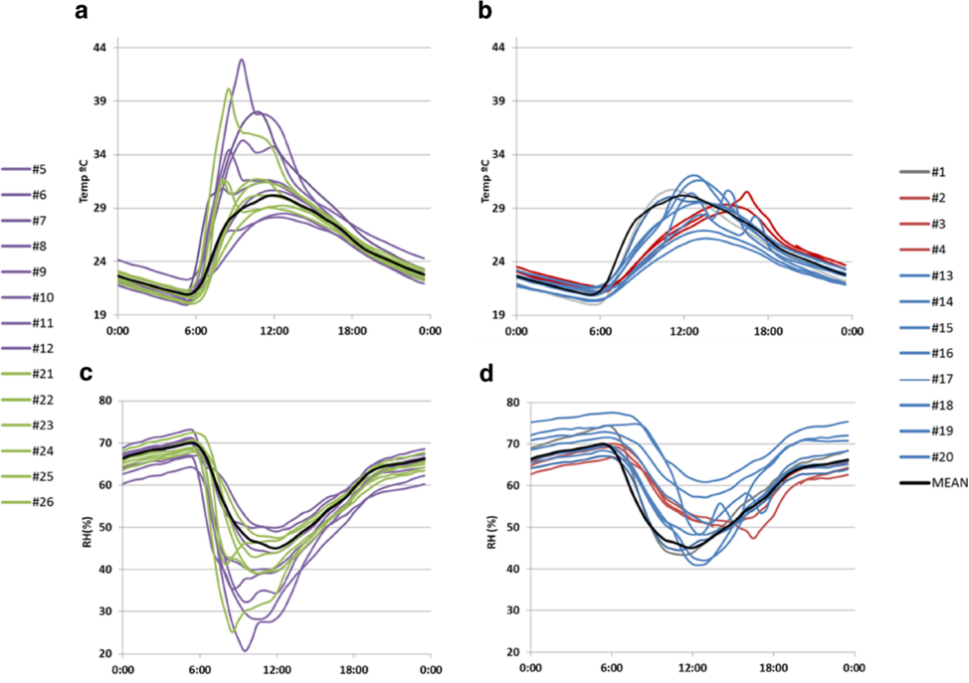
**Mean daily trajectories of temperature and RH in summer. a**) Temperature at rooms 1 and 3; **b**) temperature at rooms 2 and 4; **c**) RH at rooms 1 and 3; **d**) RH at rooms 2 and 4. Summer period: 7/23/2008 to 9/20/2008. Color codes indicate the location of probes: room 1 (green), room 2 (blue), room 3 (violet), room 4 (red). The thicker black line corresponds to the average time series of all probes.

As the daily thermohygrometric variations are more prominent in summer, it was decided to check firstly the data recorded from July 23^rd^ until September 22^nd^ 2008 in order to better appreciate the differences among trajectories. After discarding the abnormal values caused by incident sunlight, we calculated the average temperature recorded in summer by data-logger #1 at 0:00, 0:30, 1:00… and so on until 12:00 PM, resulting a daily mean time series. The same procedure was applied to all data-loggers. Daily trajectories of RH were also obtained (Figure 9).

Daily variations of temperature are more pronounced in rooms 1 and 3 (Figure 9a). Ceramic tiles protecting room 4 reduce the amplitude of the daily cycles (Figure 9b), which clearly indicates that this type of roof seems more convenient for the conservation of frescoes than the transparent polycarbonate sheets covering the other rooms. Actually, marked oscillations of thermohydrometric parameters represent a serious risk for the long-term preservation of frescoes.

All temperature trajectories seem to converge at night to a common pattern, but their variability is much higher at midday, with a range of about 15°C comparing the values of #9 *vs*. #11. Moreover, the shape of these trajectories differs at midday as the maximum value is reached around 9:00 AM in some cases (e.g. #8, #9 or #21) while other probes registered the maximum temperature about 12:00 AM (e.g. #13, #14 or #20). In the case of RH, the highest variability is observed at about 10:00 AM. At this time, the range among daily RH trajectories is about 45% (#21 *vs*. #25).

### Analysis of variance (ANOVA)

Different statistical parameters were calculated for each day and each temperature data-logger: minimum (T_min_), maximum (T_max_) and average. Similarly, daily averages of RH, minimum (RH_min_) and maximum (RH_max_) values were also obtained. In order to study the effect of height and wall orientation where the probe was located, different ANOVA models were carried out with these parameters. In all cases, two factors were considered: day and data-logger. The effect of day is not of interest here, and results will only be discussed according to the second factor. The best results were obtained with RH recorded in summer (from 7/24/2008 to 9/22/2008). Alternative ANOVAs using only winter data or both periods were also checked, but the interpretation was less clear.

**Figure 10:**
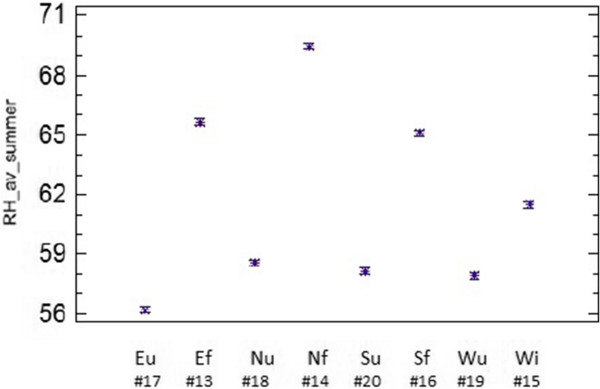
**ANOVA results (room 2).** ANOVA results showing the differences among probes in room 2 on the daily mean RH recorded in summer 2008. For each data-logger, the average and 95% LSD interval is depicted. Codes indicate the wall orientation where each sensor is facing (N: north; S: south, E: east, W: west) and the height: f (floor level), u (upper) or i (intermediate).

Regarding room 2, the daily average RH in summer was higher in data-loggers on the floor than those hanging on the walls at about 3 m of height. LSD (Least Significant Difference) intervals of sensors on the ground (#13, #14 and #16) do not overlap with LSD intervals of those at the upper position (#17, #18, #19 and #20) (Figure 10), which indicates that differences are statistically significant (α=0.05). Such significant differences of RH with respect to sensor height are not so apparent in the other lodgings. We hypothesize that the reason could be the different microclimate in room 2 given that it is the only one delimited by four walls, which probably provides more stable ambient conditions. This result suggests that sensor height is more important than wall orientation for monitoring semi-confined environments with similar characteristics as room 2. The mean RH of wall probes #18, #19 and #20 was nearly the same (Figure 10). By contrast, the differences among floor sensors resulted statistically significant: mean RH recorded by the north-oriented probe (#14) were higher than in the case of those oriented to the east (#13) or south (#16). Sensor #15 remains at 30 cm from the ground which is an intermediate position and, remarkably, the average RH recordings of this data-logger are also intermediate (Figure 10).

**Figure 11:**
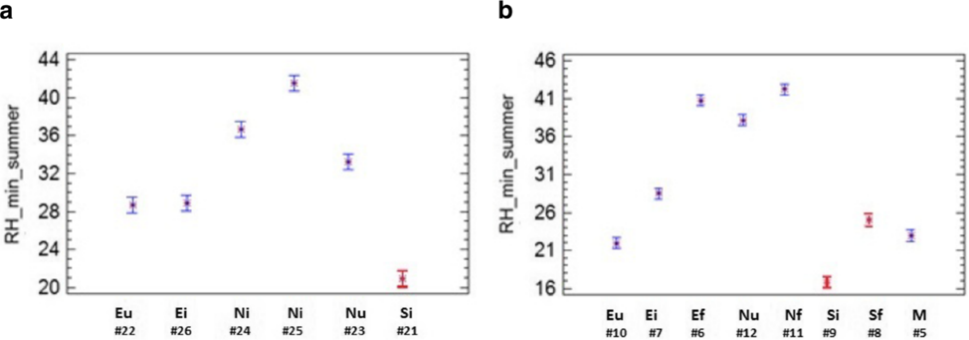
**ANOVA results (rooms 1 and 3).** ANOVA results (average and 95% LSD intervals) showing the differences among probes in room 1 (**a**) and 3 (**b**) on the daily minimum RH recorded in summer 2008. The codes indicate the wall orientation (N: north, S: south, E: east, W: west, and M: mosaic) and the height: f (floor level), i (intermediate) or u (upper).

**Figure 12:**
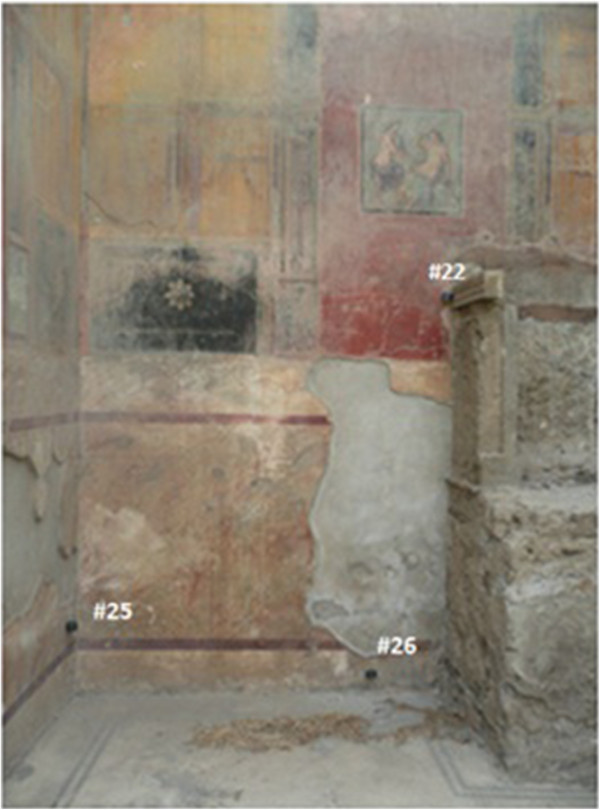
**Mural frescoes in Ariadne’s house (western wall of room 1).** Three probes can be observed: #22, #25 and #26, which remain at 240, 54 and 15 cm from the floor, respectively.

Rooms 1 and 3 present a similar size and orientation (Figure 1), both are comprised by three walls and are roofed with transparent polycarbonate sheets. Figure 11 shows that probes facing to the south in both rooms (LSD intervals highlighted in red) tend to present lower RH_min_ values than the rest. Conversely, north-oriented sensors in room 1 (#23, #24 and #25) recorded significantly higher RH_min_ values than east-oriented data-loggers (#22 and #26). This outcome is intuitively appealing because the south orientation receives more sunlight along the day. The significant differences between probes #25 and #26 were somewhat unexpected because they are separated just 2 meters away (Figure 12). The effect of orientation cannot be studied in room 4 because all sensors were basically located on the same semicircular wall.

An additional effect of sensor height is also apparent in room 3 (Figure 11b). Actually, comparing probes with the same orientation, those on the floor recorded significantly higher RH_min_ values. Results reported here indicate that data-loggers located on the ground have a different pattern, and consequently all of them will be treated as an independent cluster in the bivariate plots presented in the next section.

Different ANOVAs were also performed with daily average temperatures, T_max_ and T_min_, but results did not provide a clear interpretation regarding the effect of height and wall orientation on these parameters. Further studies with multivariate statistical methods will be necessary for this purpose. The fact that RH provides more information than temperatures was also found in a recent study [21].

Summarizing, sensor height (floor *vs.* upper position) was the main cause of RH variability in the room with four walls. An additional effect of wall orientation on RH_min_ was observed in rooms 1 and 3 that are quite similar. Consequently, it appears that the location of probes was appropriate, and similar criteria should be applied in further studies of outdoor monitoring. In closed rooms, it seems advisable to install sensors on the floor, and two different orientations might be enough for wall sensors. By contrast, in outdoor sites or lodgings with a high rate of air exchange, special care should be taken in wall orientation. In all cases, direct sunlight on data-loggers should always be avoided to prevent abnormal measurements.

### Bivariate plots

**Figure 13:**
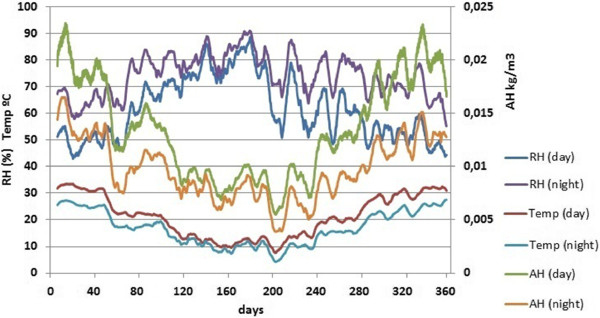
**Evolution along the year of daily mean parameters: temperature, AH and RH.** Data recorded by all probes during the daytime (8:00 AM to 8:00 PM) and night-time (8:00 PM to 8:00 AM). Day 0 corresponds to 24^th^ July 2008 (days <60: summer; 60–151: autumn; 152–239: winter; 240–331: spring). Trajectories were smoothed using a moving average with a weekly window size.

Figure 13 shows that summer is characterized by more pronounced thermohygrometric differences between day and night. Moreover, mean temperatures are higher in summer while the opposite applies to RH. As a result, both parameters are negatively correlated. Marked cycles of RH which occur along the year are also reflected. Winds like sirocco, tramontane and episodes of hot and persistent humid air are typically recorded in the Mediterranean basin. This work deals with a long-term monitoring (372 days), therefore the observed results are an average of the different wind effects along this period, avoiding their influence on the daily cycles and on the correlation between RH and temperature.

**Figure 14:**
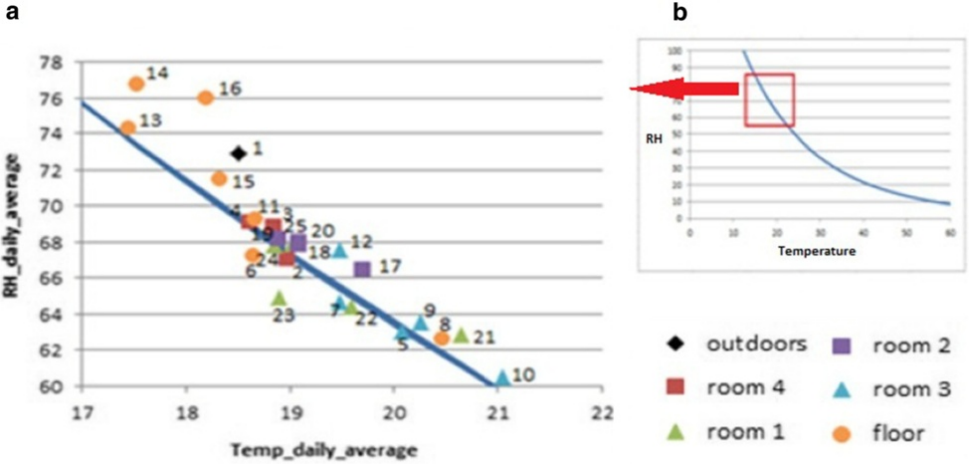
**Bivariate plot of daily average RH *vs*. temperature. a**) Bivariate plot of daily average RH from each probe with respect to daily average temperature, corresponding to the year 2008–2009 (372 days). **b**) Theoretical function relating RH and temperature (Equation (2)), which is also depicted inside the bivariate plot.

RH is defined as the partial pressure of water vapor in the air-water mixture divided by the saturated vapor pressure. At higher temperatures, the air can hold more water prior to reaching the saturation, which may partly explain the negative correlation between RH and temperature (r = −0.862) observed in Figure 14a. Attempting to further describe the thermohygrometric variations along the year, mean values of absolute humidity (AH) are also depicted in Figure 13. They were calculated using Equation (1) [31] according to RH (%) and temperature (°C).

(1) $$AH=RH\cdot \frac{0.0132295}{273.16+T}\cdot \exp\left(\frac{17.2694\cdot T}{238.3+T}\right)$$

(2) $$RH=0.0109\cdot \frac{273.16+T}{0.0132295}\cdot \exp\left(-\frac{17.2694\cdot T}{238.3+T}\right)$$

In order to characterize the dissimilarities among probes, different bivariate plots were obtained and visually inspected. Figure 14a displays average RH *vs*. average temperatures. Taking into account the relationship between RH and AH (Equation (1)), RH was plotted as a function of temperature inside this figure by considering AH=0.0109 kg/m^3^ (Equation (2)). This value is the one that achieves the best goodness-of-fit in Figure 14a and it was estimated using non-linear regression after disregarding floor probes and #1.

**Figure 15:**
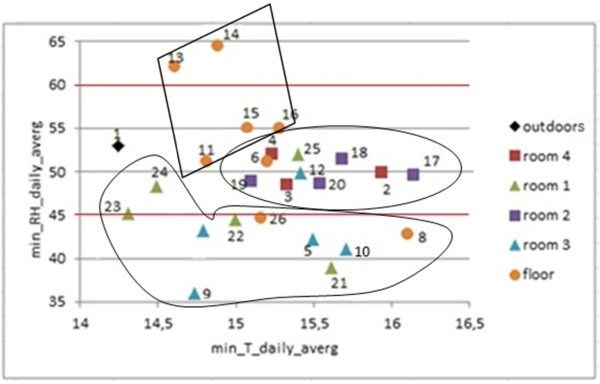
**Bivariate plot of daily minimum RH *vs*. temperature.** Bivariate plot of daily minimum RH from each probe *vs*. daily minimum temperature, averaged for all the 372 days. Horizontal lines in red (45–60%) correspond to the range of RH recommended by the standard DM 10/2001 for the conservation of mural paintings.

Bivariate plots of T_max_, T_min_, RH_max_ and RH_min_ are also of interest to illustrate the differences among recorded trajectories. The comparison of RH_min_*vs*. T_min_ (Figure 15) does not show any correlation, but the plot reveals three clusters of probes according to their location: (i) floor, (ii) rooms 1 and 3, and (iii) rooms 2 and 4. Minimum temperatures tended to be cooler at the floor level. The bivariate plot of RH_max_*vs*. T_max_ (Figure not shown) was also visually inspected, but clusters are better discriminated in Figure 15.

**Figure 16:**
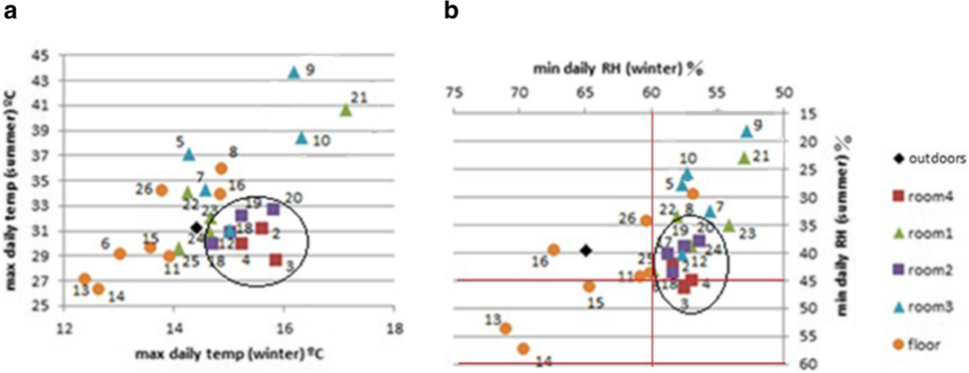
**Bivariate plots (summer *vs*. winter). a**) Bivariate plot of daily maximum temperature (T_max_) averaged for the summer period (7/24/2008 – 9/22/2008) *vs*. T_max_ averaged for the winter period (12/21/2008 – 3/31/2009). **b**) Bivariate plot of daily minimum RH (RH_min_) in summer *vs*. RH_min_ in winter. Axes were inverted in the right plot to ease the comparison. Lines in red (45–60%) correspond to the range of RH recommended by the standard DM 10/2001.

Additional bivariate plots considering only the summer or winter periods were also inspected. The most interesting ones that reflect the different ambient conditions between the two seasons correspond to T_max_ (Figure 16a) and RH_min_ (Figure 16b). Sensors in rooms 2 and 4 are highlighted with an ellipse. The relative position of probes in both figures is very similar given the tight correlation between RH and temperature, which reveals certain redundant information. Based on this correlation, it is not justified to use the same number of data-loggers for recording both parameters in open-air archaeological sites.

According to Figures 14, 15 and 16 probes on the floor recorded higher RH and lower temperatures. In particular, most of them yielded the highest mean RH along the year (Figure 14a), and the highest RH_min_ values in winter (Figure 16b). Moreover, they yield the lowest mean temperatures along the year (Figure 14a) and the lowest T_max_ in winter (Figure 16a). As an exception, probe #8 registered much higher temperatures (Figure 14a). The reason is unclear, but taking into account that #8 was oriented to the south, perhaps it received more diffuse solar radiation. The same reason might explain why probes #9 and #21 recorded the highest T_max_ (Figure 16a) and the lowest RH_min_ values (Figures 11 and 16b), given that they were also south-oriented. This hypothesis is mere speculation and further studies using more data-loggers will be necessary to investigate this issue.

Assuming that AH is constant inside all rooms, the curve depicted in Figure 14a highlights that floor probes #14 and #16 (room 2) deviate from the theoretical relationship between RH and temperature. The RH mean daily time series of both probes is also atypical (Figure 9d) as the daily decrease of RH registered at sunrise is delayed in about 30 min with respect to the other trajectories. This abnormal performance might be caused by diffusion of water vapor by capillarity through the ground to the boundary layer in contact with the data-logger. The fact that higher RH_min_ values were recorded by floor sensors is also consistent with this hypothesis. Room 2 is the only one comprised by four walls, but it is uncertain if this issue could also explain their atypical performance.

Probe #1 also appears as an outlier in Figure 14, which suggests that AH outside Ariadne’s house might be higher than indoors. Moreover, this probe recorded the lowest T_min_ (Figure 15) and higher RH_min_ values in winter with respect to wall sensors (Figure 16b). Results reveal that the microclimate reflected by #1 was slightly different, which suggests using one or more outside sensors in similar monitoring studies.

According to DM 10/2001 [30], the recommended interval of RH is 45-60%. Regarding RH_min_, only probes in room 4 satisfy this condition in summer and winter (Figure 16b). By contrast, RH_min_ tends to be lower than 45% in rooms 1 and 3 (Figure 15), which indicates ambient conditions too dry for an appropriate conservation of frescoes particularly in summer.

Figures 14, 15, 16 reveal that ambient conditions in rooms 2 and 4 were cooler in summer, more humid and more stable (i.e., with less variability among probes) than in rooms 1 and 3. In winter, temperatures in rooms 2 and 4 tended to be less cold (Figure 16a), which appears more appropriate for preserving the frescoes. The observed differences among lodgings can be explained by the roof type and number of walls of the room. Actually, room 2 is characterized by an environment more isolated from outdoor conditions and room 4 is partly covered with ceramic tiles that prevent incoming sunlight. The others are roofed with transparent sheets that produce a greenhouse effect, which tends to increase the temperature. Thus, opaque shelters seem more convenient. Taking into account that probe #5 was located under a glass, the greenhouse effect could also explain the higher temperatures recorded by this data-logger with respect to the others on the floor of room 3 (Figure 14a).

Experts in restoration of frescoes are currently assessing the conservation state of paintings in Ariadne’s house. Preliminary results suggest that frescoes in room 2 are better preserved that the rest, which is of interest because room 2 presents ambient conditions closer to the range recommended by DM 10/2001 [30]. This issue supports the use of this standard for discussing the statistical data analysis.

Given the unfavorable environment revealed in the present study, the transparent roof in rooms 1–3 was replaced in December 2009 by undulating roof sheets made of fiber cement with a thickness of about 5 mm. The conclusion that transparent roofs are inappropriate for the conservation of outdoor wall paintings might seem obvious for experts in the field, but the competing authorities requested empirical evidence prior to approving the roof change. The effectiveness of this corrective measure will be assessed in a future study.

## Conclusions

The statistical analysis of RH and temperatures recorded in Ariadne’s house has revealed that autonomous data-loggers with a sampling rate of two readings per hour seem appropriate. Considering that few works have monitored microclimate environments of outdoor or semi-confined archaeological sites, results reported here are of interest for similar studies regarding the number, type and position of data-loggers, as well as for the subsequent statistical data analysis. The first step of the analysis should be the bias correction of all measurements based on the calibration experiment.

Descriptive tools have been effective for highlighting the dissimilarities among probes. The mean daily trajectories displayed in Figure 9 provide useful information about the difference in average values and shapes among data-loggers. This type of plot is used in several fields of science to visualize and synthesize differences in recorded values, but it is rarely applied in microclimate monitoring where trajectories of a few days randomly chosen are usually presented.

By means of ANOVA and bivariate plots, it was found an effect of roof type, wall orientation and sensor height on microclimate conditions. Higher RH values were recorded by data-loggers on the floor, and the south orientation was characterized by lower RH values because it tended to receive more sunlight radiation. The differences among probes suggest that at least four data-loggers per room would be required in similar studies to assess the effect of different factors and to detect abnormal trajectories. One or more control sensors are also recommended. Regarding the number of walls of the room, rooms with four walls will require less number of sensors at wall levels, where two different orientations might be enough. At floor level, north orientation and a complementary one (south or east) should also be monitored. If several lodgings present similar characteristics, orientation, size and number of walls, it seems sufficient to monitor just one of them.

A tight correlation was found between average RH and temperatures, which suggests that it is not necessary to use the same number of RH and temperature sensors in open-air archeological sites. Redundant information will also result if lodgings with very similar characteristic are monitored, which reveals the importance of choosing properly the right location for probes.

Results reveal that summer conditions in Pompeii are more unfavorable for the conservation of frescoes according to the standard DM 10/2001 because the microclimate was too dry and hot. Consequently, ambient conditions in similar studies should be basically monitored in summer. Transparent roofs produce a greenhouse effect that increases the temperature, which is undesirable. Based on the results, corrective measures were taken by replacing the type of roof. The effectiveness of such measures will be assessed in future studies.

## Methods

### Installation of data-loggers

**Figure 17:**
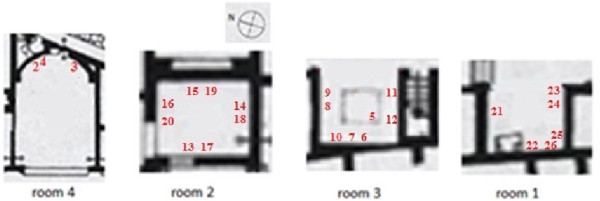
**Position of thermohygrometric probes.** Room code as in Figure 1. Probe #5 was installed at the center of room 3 under a glass box on the ground protecting a mosaic. Probes #6, #8 and #11 were located on the floor level under a ceramic tile that was placed to protect them from accidental stepping.

The monitoring study started on July 23^rd^ 2008 when 25 probes were installed in the four rooms under investigation (exact location shown in Figure 17). One additional device (#1 in Figure 1) was placed oriented to the east on the top of an outside wall and it was covered with a ceramic tile to protect it against rainwater and sunlight. Data recorded by #1 cannot be regarded as reference outdoor conditions like those of a meteorological station (due to the specific policy required for their installation). However, in this work #1 is used as a reference considering its limitations when analyzing the results.

Information about the position of probes (room and wall orientation) is indicated in Figure 17. Table 1 shows the height with respect to floor level.

One target was to study the vertical gradient of RH and temperature. For this purpose, data-loggers were placed in the four rooms at three levels: floor, intermediate (15 – 200 cm) and upper position (200 – 338 cm). Thermographic studies carried out previously [11] were also taken into consideration to decide the best location of probes. The intermediate position was omitted in room 2 because it is more isolated from the outside environment and, hence, lower thermohygrometric gradients were expected. Data-loggers in room 4 were not located on the ground because the floor was full of rubble, which might result in microclimate conditions not directly comparable with respect to the floor of the other rooms.

Probe #5 was placed inside a glass box that protects a mosaic on the floor of room 3 in order to assess the microclimate inside this box. All probes on the ground except #5 were placed near the walls (Figure 17), as otherwise they would hamper the free movement of visitors inside the room. The rest were installed hanging on walls except the reference probe #1.

**Figure 18:**
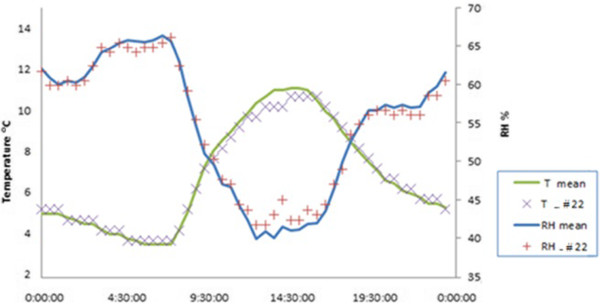
**Daily time series of probe #22.** Temperature and RH values registered by one specific probe (#22) during a day randomly chosen (February 16^th^ 2009) as well as average values collected by the 26 probes. One measurement was recorded every 30 min. According to the manufacturer, the accuracy is ±0.5°C and ±5% for temperature and RH data-loggers, respectively.

All data-loggers were programmed to register two recordings per hour. Figure 18 shows that the daily cycles of temperature and RH are clearly reflected by the data of probe #22 chosen as example, which suggests that a sampling rate of one measurement every 30 minutes seems convenient.

### Calibration of sensors

A calibration experiment is another compulsory step in a methodology of microclimate monitoring. Two different calibration experiments were conducted. The first was carried out in two periods (at the beginning and at the end of the experiment) in order to study if measurements from one or more sensors were biased with respect to the average recorded by all sensors. During both periods, all data-loggers were kept in a small compartment in contact with the indoor air of the laboratory whose conditions varied during the experiment.

For this calibration experiment, all data-loggers were confined inside an 8-liter compartment (20×10×40 cm) that was not sealed and allowed the inside air to reach an equilibrium of RH and temperature with respect to the outside. This experiment was carried out during a 3-day period (07/18/2008 to 07/21/2008) prior to the installation of probes in Ariadne’s house and during 28 days after the monitoring study (07/31/2009 to 08/28/2009), once all data-loggers were back in the laboratory. In the first calibration period, temperature ranged from 27.5°C to 29.5°C and RH ranged from 49% to 60%. In the second period, the range of temperature was 21.5 – 26°C, and 58 – 82% in the case of RH.

A further experiment was carried out with aqueous solutions of two salts (lithium chloride and sodium chloride) according to the standard ASTM E 104–02 [32] in order to study the measurement errors of RH data-loggers.

### Frequency of data recording

The monitoring study started on July 23^rd^ 2008 and ended on July 30^th^ 2009, resulting a period of 372 days. All data-loggers were programmed to register one measurement every 30 minutes, which implies 1,440 recorded values per month (i.e., 30 days × 24 hours/day × 2 data/hour). Taking into account that these devices are able to store 8,192 registries, it turned out that they could operate for about 5.7 months, which allowed the scientific team to travel conveniently from Spain to Pompeii three times a year to collect the data.

### Analysis of variance (ANOVA)

Taking into account that similarities and dissimilarities among trajectories might be different in summer *versus* winter, a summer period of 61 days was considered from 7/24/2008 to 9/22/2008 (i.e., from the first day of monitoring until the autumnal equinox). Similarly, a winter period of 90 days was considered from 12/21/2008 (winter solstice) to 31/3/2009. For each day and each data-logger, different statistical parameters of RH and temperature were calculated: daily averages, maximum and minimum values. These data were arranged in a matrix comprised by 3,926 rows (i.e., 61+90 days × 26 data-loggers). Four factors were considered and included in the matrix: day, season (summer or winter), data-logger code and room code. In order to study if the differences among probes in the same room were statistically significant, multifactor ANOVAs were carried out considering two factors (day and data-logger) and selecting data recorded in summer in one room. Next, the analysis was repeated for each room and considering only the summer or winter periods. All ANOVAs were performed using the software Statgraphics 5.1 [33].

## Competing interests

The authors declare that they have no competing interests.

## Authors’ contributions

All the authors contributed equally to this work. PM is developing her PhD thesis under a predoctoral grant focused on the statistical monitoring for preventive conservation of cultural heritage; FJGD was responsible for the design and implementation of the monitoring system, and MZ contributed with the statistical analyses. All authors read and approved the final manuscript.
